# Human Chorionic Gonadotropin (hCG)-Induced Remodeling of the Granulosa Cell Exosomal Proteome: Implications for Follicular Communication

**DOI:** 10.3390/cells15110956

**Published:** 2026-05-22

**Authors:** Francesca Mancini, Michela Cicchinelli, Emanuela Teveroni, Erica Pazzaglia, Donatella Lucchetti, Giulia Artemi, Valentina Palmieri, Federica Iavarone, Domenico Milardi, Andrea Urbani, Tullio Ghi, Annamaria Merola, Fiorella Di Nicuolo

**Affiliations:** 1 International Scientific Institute Paul VI, Fondazione Policlinico Universitario A. Gemelli IRCCS, 00168 Rome, Italy; francesca.mancini@policlinicogemelli.it (F.M.); emanuela.teveroni@guest.policlinicogemelli.it (E.T.); 2 Clinical Chemistry, Biochemistry and Molecular Biology Operations (UOC), Fondazione Policlinico Universitario A. Gemelli IRCCS, 00168 Rome, Italy; federica.iavarone@unicatt.it (F.I.); andrea.urbani@policlinicogemelli.it (A.U.); 3 Department of Basic Biotechnological Sciences, Intensivological and Perioperative Clinics, Università Cattolica del Sacro Cuore, 00168 Rome, Italy; michela.cicchinelli@unicatt.it; 4 Multiplex Spatial Profiling Facility, Fondazione Policlinico Universitario A. Gemelli IRCCS, 00168 Rome, Italy; erica.pazzaglia@unicatt.it (E.P.); donatella.lucchetti@unicatt.it (D.L.); 5 Department of Translational Medicine and Surgery, Università Cattolica del Sacro Cuore, 00168 Rome, Italy; domenico.milardi@policlinicogemelli.it; 6 Department of Neuroscience, Section of Biophysics, Università Cattolica del Sacro Cuore, 00168 Rome, Italy; giulia.artemi@unicatt.it; 7 Institute for Complex Systems, National Research Council, 00185 Rome, Italy; valentina.palmieri@cnr.it; 8 Complex Operative Unit of Internal Medicine, Endocrinology and Diabetology, Department of Translational Medicine and Surgery, Fondazione Policlinico Universitario A. Gemelli IRCCS, 00168 Rome, Italy; 9 Department of Women, Child and Public Health, Fondazione Policlinico Universitario A. Gemelli IRCCS, 00168 Rome, Italy; tullio.ghi@policlinicogemelli.it (T.G.); annamaria.merola@policlinicogemelli.it (A.M.); 10 Faculty of Medicine and Surgery, Catholic University of the Sacred Heart, 00168 Rome, Italy

**Keywords:** human granulosa cells (KGN), human chorionic gonadotropin (hCG), extracellular vesicles (EVs), exosomes, integrin α3 (ITGα3), Galectin-3-binding protein (LGALS3BP)

## Abstract

Human follicular development depends on coordinated communication between granulosa cells (GCs) and oocytes through endocrine cues, direct contacts, and extracellular vesicles (EVs). Exosomes are key EV mediators of intrafollicular signaling, but their cargo and functions in gonadotropin-stimulated GCs remain poorly defined. The human granulosa-like tumor cell line KGN was used to investigate exosome secretion and protein composition following human chorionic gonadotropin (hCG) stimulation. Exosomes were isolated by ultracentrifugation, characterized via nanoparticle tracking analysis (NTA), Scanning Electron Microscopy (SEM) and Western blotting, and analyzed using high-resolution mass spectrometry. Comparative proteomics integrating exosomal profiles with the whole secretome were performed, followed by bioinformatic analyses of protein networks, gene ontology, and pathway enrichment. hCG reshaped exosomal cargo, identifying 59 proteins enriched in exosomes, including Integrin α3 (ITGα3), Galectin-3-binding protein (LGALS3BP), tetraspanins (CD63, CD151), and proteasome subunits. Functional enrichment indicated roles in extracellular matrix remodeling, integrin signaling, proteostasis, and steroidogenesis. Comparison with the secretome revealed distinct protein distributions, supporting selective exosomal packaging. Western blot confirmed increased ITGα3 and LGALS3BP levels in exosomes upon hCG treatment. In conclusion, hCG modulates exosome cargo composition in granulosa cells, uncovering a novel mechanism of extracellular regulation.

## 1. Introduction

Successful follicular development depends on tightly orchestrated cross-talk between somatic and germ cells, which is crucial for oocyte growth, meiotic progression, and ovulation [1]. This bidirectional communication is mediated by endocrine and paracrine factors as well as direct cell–cell interactions, including gap junctions and transzonal projections [2,3]. Within this regulatory network, granulosa cell maturation represents a central event, as it ensures appropriate follicular growth and the acquisition of oocyte developmental competence. This process is highly controlled by gonadotropic stimulation: follicle-stimulating hormone (FSH) primarily supports granulosa cell proliferation and differentiation, whereas luteinizing hormone (LH) initiates the cascade of molecular and cellular events that ultimately leads to ovulation [4,5].

Although FSH and LH physiologically drive final follicular maturation, human chorionic gonadotropin (hCG) is commonly employed in clinical settings to replicate the preovulatory LH surge, especially in ovulation induction and luteal phase support [6]. This is due to its high affinity for the LH receptor and its longer half-life, which results in more sustained receptor activation [6].

Through activation of LH/CG receptors expressed on granulosa and theca cells, hCG initiates key ovulatory signaling cascades, promoting the transcription of critical regulators of ovulation, including the progesterone receptor (PGR), enzymes involved in prostaglandin synthesis, and epidermal growth factor-like ligands such as Amphiregulin (AREG), Epiregulin (EREG), and Betacellulin (BTC) [7,8,9]. These molecular events stimulate progesterone and prostaglandin production, drive cumulus cell expansion, and ultimately culminate in follicular rupture. In addition, hCG enhances the release of paracrine mediators, including neurotensin, which further contribute to the fine regulation of ovulatory processes [10].

In a recent study, our group characterized the gonadotropin-stimulated granulosa cells secretome by a proteomic approach. This human granulosa-like tumor cell line (KGN) is a widely used in vitro model that preserves essential characteristics of granulosa cells, including steroidogenic capacity [11]. Proteomic analysis demonstrated substantial changes in proteins associated with extracellular matrix organization, steroid biosynthesis, and cytoskeletal dynamics, alongside a pronounced enrichment in components of the semaphorin signaling pathway [12]. It has been demonstrated that within the ovary, semaphorins and their receptors—such as plexins and neuropilins—are dynamically expressed in granulosa cells, theca cells, and oocytes, where they contribute to the regulation of follicular growth [13,14]. Although these findings enhance our understanding of the molecular networks involved in follicular development and point to potential biomarkers for predicting ovarian responsiveness to gonadotropin stimulation, the human ovulatory cascade remains incompletely characterized.

Growing evidence indicates that extracellular vesicles (EVs) present in follicular fluid constitute a key mechanism of intrafollicular communication, contributing to the regulation of genes involved in follicular growth, and ovulation [15,16].

EVs represent a heterogeneous group of membrane-enclosed particles, delimited by a lipid bilayer, that are secreted by nearly all cell types [17]. They function as intercellular messengers by delivering bioactive cargo—such as proteins, lipids, and nucleic acids—from donor to recipient cells [18,19].

Based on their origin and size, EVs are commonly classified into exosomes, microvesicles, and apoptotic bodies. Exosomes (30–150 nm) arise within multivesicular bodies and are secreted upon fusion of these compartments with the plasma membrane [19,20]. In contrast, microvesicles (50–1000 nm) originate through outward blebbing of the plasma membrane [19,21], while apoptotic bodies (200–5000 nm) are produced during the process of programmed cell death. [17]. EV populations are often characterized by the presence of membrane-associated proteins such as the tetraspanins CD9, CD63, and CD81 although universally accepted EV-specific markers have yet to be established [22,23].

Among these EVs, exosomes have been identified in human follicular fluid as well as within the perivitelline space surrounding the oocyte [24]. By mediating the transfer of several bioactive molecules between follicular cells and the oocyte, particularly mRNAs, miRNAs, and proteins, exosomes contribute to the dynamic exchange of signals required for synchronized follicle growth and developmental competence [24,25]. These findings underscore the relevance of EV-mediated signaling in maintaining ovarian function and reproductive success.

This study aimed to characterize exosomes isolated from the conditioned medium of KGN cells and to determine the effect of hCG stimulation on their protein cargo composition. A qualitative proteomic approach was applied to evaluate molecular changes within these extracellular vesicles, followed by an integrated comparative analysis combining exosome profiling data with hCG-induced secretome findings.

## 2. Materials and Methods

### 2.1. Cell Culture and Exosomes Isolation

The study employed the human granulosa-like tumor cell line KGN (kindly provided by Prof. D. Gallo), which retains key functional features of normal ovarian granulosa cells, such as gonadotropin receptor expression and steroidogenic capacity [11]. Cells were cultured and routinely passaged in phenol red-free DMEM/F12 medium supplemented with 10% charcoal-stripped fetal calf serum, L-glutamine, non-essential amino acids, and penicillin/streptomycin (Thermo Fisher Scientific, Waltham, MA, USA). Cultures were maintained at 37 °C in a humidified incubator with 5% CO_2_. For experimental treatments, KGN cells were plated and grown to approximately 80% confluence, rinsed with pre-warmed PBS, and transferred to serum-free medium for 24 h to induce cellular starvation. The starvation medium was then replaced with serum-free DMEM/F12 containing antibiotics and human chorionic gonadotropin (hCG; Gonasi HP^®^, IBSA Farmaceutici, Lodi, Italy) at a final concentration of 1.0 IU/mL. After 48 h of stimulation, culture supernatants were collected for the isolation of KGN-derived extracellular vesicles (EVs) and subjected to sequential centrifugation steps to eliminate cell debris and non-specific contaminants. The differential ultracentrifugation method provides an enriched population of small extracellular vesicles/exosomes.

The concentration of hCG (1.0 IU/mL) was selected based on previous studies demonstrating that this range effectively activates LH/hCG receptor signaling in granulosa cells without inducing non-physiological effects [9,12].

The 48 h treatment duration was chosen to allow sufficient accumulation of secreted proteins and extracellular vesicles, as commonly reported in similar in vitro models [25].

Briefly, the EV-enriched conditioned cell culture media were first centrifuged at 750× *g* for 15 min, followed by a second centrifugation at 1500× *g* for 5 min. The resulting supernatants were collected and centrifuged at 17,000× *g* for 45 min. The supernatants were then transferred to fresh tubes and subjected to ultracentrifugation at 120,000× *g* for 2 h. The EV-enriched pellets were resuspended in PBS filtered through a 0.1 µm filter and used for further characterization, as described below, as well as for the preparation of protein extracts for Western blot analysis (EV-associated markers CD9 and CD63 and negative marker Calnexin). Quantification of EVs was performed using the Bradford assay.

Vesicle size distribution and concentration were assessed by a nanoparticle tracking analysis (NTA) NS300 instrument (Malvern Panalytical Ltd., Malvern, UK) with NTA software version 3.4 (build 3.4.4). Silica microspheres of 100 nm were routinely measured to verify proper instrument performance. For NTA, EV samples were diluted 1:100–1:500 in 1 mL of PBS. The EV preparations were then loaded into the sample chamber and analyzed according to the following measurement script: initialization, 5 s delay, and 60 s acquisition. Measurements were performed twice for each sample. The camera gain was set to level 15 for all measurements, and data were analyzed using proprietary software (version 3.4). Purified KGN-derived EVs were finally stored at −80 °C until proteomic profiling.

For Scanning Electron Microscopy (SEM) imaging, samples were fixed, dehydrated and sputter coated as previously reported [26]. Briefly, glutaraldehyde (2.5%) was used to fix the vesicles for 20 min. The samples were then dehydrated with ethanol directly on conductive carbon tape on SEM stubs. After air drying, the samples were sputter-coated with platinum. Micrographs were acquired with SEM Supra 25 (Zeiss, Oberkochen, Germany) at several magnifications (scale bars are reported on each image). Vesicle diameters were analyzed using FIJI software (version 1.54f; National Institute of Health, Bethesda, MD, USA).

### 2.2. Enzymatic Digestion and Mass Spectrometry Analysis

Protein processing was carried out using the filter-aided sample preparation (FASP) approach, which enables simultaneous protein cleanup and enzymatic digestion [27]. For each sample, 50 μg of protein was subjected to reduction with 8 mM dithiothreitol (DTT) in urea buffer (8 M urea, 100 mM Tris), followed by alkylation with 50 mM iodoacetamide (IAA) in the same buffer. Proteolytic digestion was then performed on Microcon^®^ centrifugal filter units (Merck Millipore Ltd., Cork, Ireland) using trypsin at a final concentration of 1 μg/μL.

Peptide mixtures were analyzed by a bottom-up proteomics workflow using an UltiMate™ 3000 RSLCnano UHPLC system (Thermo Fisher Scientific, Waltham, MA, USA) coupled to an Orbitrap Fusion Lumos Tribrid mass spectrometer (Thermo Fisher Scientific, Waltham, MA, USA) equipped with a nano-electrospray ionization (ESI) source. Chromatographic separation was achieved on a PepMap RSLC C18 column (2 μm particle size, 100 Å pore size, 50 μm × 15 cm; Thermo Fisher Scientific, Waltham, MA, USA) using gradient elution. Mobile phase A consisted of 0.1% (*v*/*v*) formic acid in water, while mobile phase B was composed of acetonitrile/water (80:20, *v*/*v*) containing 0.1% (*v*/*v*) formic acid. The gradient program (total run time 155 min) was as follows: 3% B (0–110 min), 20% B (110–120 min), 40% B (120–125 min), 90% B (125–145 min), and re-equilibration at 3% B (145–155 min), with a constant flow rate of 0.300 μL/min.

A volume of 5 μL, corresponding to 1 μg of peptides, was injected for each run. The mass spectrometer was operated in positive ion mode using a nano-spray ionization source (spray voltage 1800 V), with the ion transfer tube maintained at 275 °C. Data acquisition was performed in data-dependent acquisition (DDA) mode, collecting high-resolution MS/MS spectra in the Orbitrap analyzer over an *m*/*z* range of 375–1500 at a resolution of 120,000, with peptide fragmentation induced by higher-energy collisional dissociation (HCD). Each sample was analyzed in triplicate. This analysis was performed using technical triplicates and does not include independent biological replicates.

Raw MS/MS data were processed using Proteome Discoverer software version 2.4.1.15 (Thermo Fisher Scientific, Waltham, MA, USA), employing the SEQUEST HT search engine against the UniProtKB/Swiss-Prot Homo sapiens protein database for qualitative “processing” and “consensus” workflows. Search parameters included a precursor mass range of 350–5000 Da, precursor mass tolerance of 10 ppm, fragment mass tolerance of 0.02 Da, up to two missed tryptic cleavages, and peptide lengths between 6 and 144 amino acids. Oxidation of methionine (+15.995 Da) was specified as a variable modification, while carbamidomethylation of cysteine (+57.021 Da) was set as a fixed modification. Peptide validation settings included false discovery rates (FDR) controlled at 1% (strict) and 5% (relaxed) and Peptide Confidence “High”. Confidence validation settings used for protein identification included false discovery rates (FDR) controlled at 1% (strict) and 5% (relaxed). To ensure robust identification, data were stringently filtered, and only proteins supported by at least two unique peptides were retained for downstream analyses.

### 2.3. Bioinformatics Analysis

Bioinformatic analyses were conducted on the mass spectrometry datasets obtained from untreated and hCG-treated KGN-derived exosomes. For qualitative assessment, proteins detected across the two samples were compiled and compared. Overlapping and sample-specific protein sets were identified by intersecting the respective protein lists, and Venn diagrams were generated to visualize shared and unique proteins between CTR and hCG conditions using the online Venn Diagram Generator tool (https://bioinformatics.psb.ugent.be/webtools/Venn/ accessed on 31 May 2025).

Pathway enrichment was performed via the Reactome database (http://reactome.org, accessed on 31 May 2025) by overrepresentation analysis based on FDR. To construct a protein–protein interaction network for the identified sperm proteins, we employed the Search Tool for the Retrieval of Interacting Genes/Proteins (STRING 12.0) software. The resulting network visually represents proteins as nodes, with edges (colored lines) connecting them to depict their interactions.

For the STRING analysis, we used a minimum required interaction score of 0.7 (high confidence) to construct the protein–protein interaction (PPI) network. The network was further filtered to include only experimentally validated and database-annotated interactions. For the Reactome pathway enrichment analysis, default settings were used, with a significance threshold of FDR < 0.05.

### 2.4. Western Immunoblotting

The expression of exosome-associated markers CD9 and CD63, as well as Calnexin, Integrin α3 (ITGα3) and LGALS3PB, were evaluated by Western blot analysis. Exosomal protein extracts were prepared from exosomes isolated from KGN cells, either untreated or exposed to 1.0 IU/mL hCG for 48 h. Briefly, exosomes were lysed using a buffer containing 0.025% NP-40 with protease inhibitors and proteins were separated by 10% SDS–PAGE under reducing conditions and subsequently transferred onto polyvinylidene difluoride (PVDF) membranes (Merck Millipore Ltd., Cork, Ireland).

EveryBlot Blocking Buffer (Bio-Rad, Hercules, CA, USA) was used to block membranes for 30 min at room temperature. The membranes were then incubated overnight at 4 °C with primary antibodies directed against CD9 (Cell Signaling Technology, Leiden, The Netherlands), CD63 (Santa Cruz Biotechnology, Inc., Heidelberg, Germany), Calnexin (Thermo Fisher, Waltham, MA, USA), LGALS3PB (Proteintech, Manchester, UK) or ITGα3 (Thermo Fisher, Waltham, MA, USA). Following incubation, membranes were washed with PBST and probed with the appropriate horseradish peroxidase (HRP)-conjugated secondary IgG antibodies diluted 1:3000 in blocking buffer. Immunoreactive signals were visualized using an enhanced chemiluminescence detection system (ECL™, Ge Healthcare, Amersham, UK) and acquired with the Alliance 2.7 chemiluminescence imaging system (UVITEC, Cambridge, UK). Densitometric analysis of the detected bands was performed using Alliance V_1607 software.

### 2.5. Statistical Analysis

Results are presented as mean ± standard deviation (SD). Statistical comparisons were carried out using one-sample *t*-test as the control group was normalized to a reference value of 1. Differences were considered statistically significant at *p* < 0.05. All statistical analyses were performed with GraphPad Prism software (version 10.3.1).

## 3. Results

### 3.1. Isolation and Identification of Exosomes

**Figure 1:**
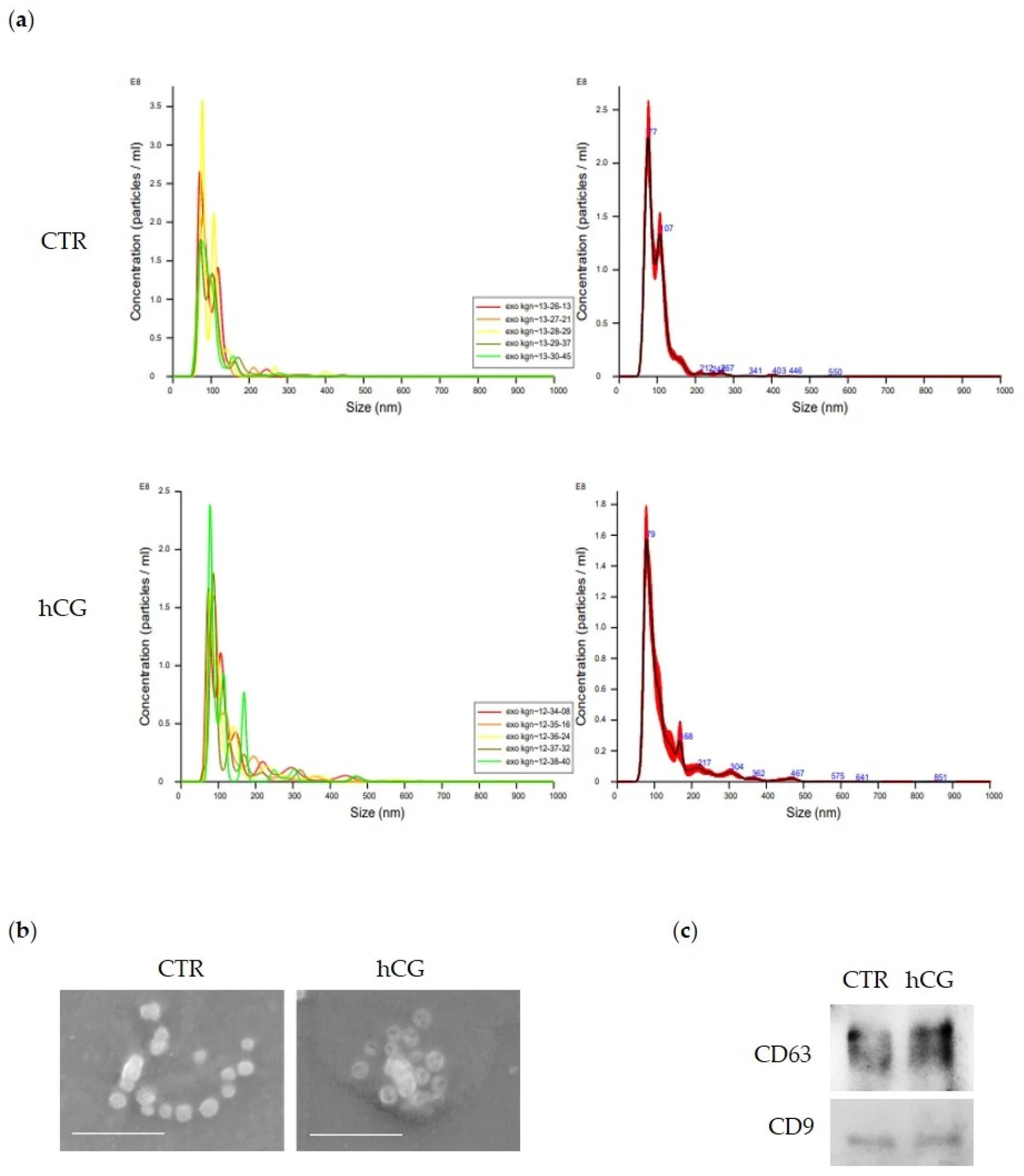
Exosome characterization. (**a**) Representative size versus concentration distribution graphs of CTR (*n* = 5), hCG (*n* = 5) EV samples analyzed by nanoparticle tracking analysis (Nanosight). (**b**) Representative SEM images of vesicles, scale bar is 1 μm. (**c**) Western blot analysis of exosomal markers CD63 and CD9 in exosome lysates from untreated (CTR) or hCG treated KGN.

Exosomes were isolated from a serum-free culture medium of KGN cells (untreated, CTR, and hCG-stimulated) and characterized by Western blot and nanoparticle tracking analysis (NTA) to confirm vesicle identity, size distribution, and concentration. Particle size distribution analysis revealed a predominant population of vesicles within the expected exosomal size range (approximately 50–150 nm) in both CTR and hCG-treated groups (Figure 1a). Particle concentrations were comparable between control and hCG-treated groups, suggesting that hCG treatment did not markedly alter total exosome yield under the experimental conditions (Figure 1a). In Figure 1b representative SEM images of the vesicles are shown. The average diameter was 95 ± 7 nm, in agreement with the NTA data. Immunoblot analysis demonstrated the presence of canonical exosomal markers in isolated vesicles (Figure 1c). Bands corresponding to established exosome-associated proteins (CD63 and CD9) were detected in both CTR and hCG-treated samples, confirming successful enrichment of small extracellular vesicles. The signal intensity appeared comparable between groups, indicating consistent isolation efficiency across conditions. Further, to confirm the identity of the exosome preparation we performed Western blot analysis of Calnexin as a negative exosome marker (Supplementary Figure S1).

### 3.2. Proteomic Analysis of Differentially Expressed Exosomal Proteins

**Figure 2:**
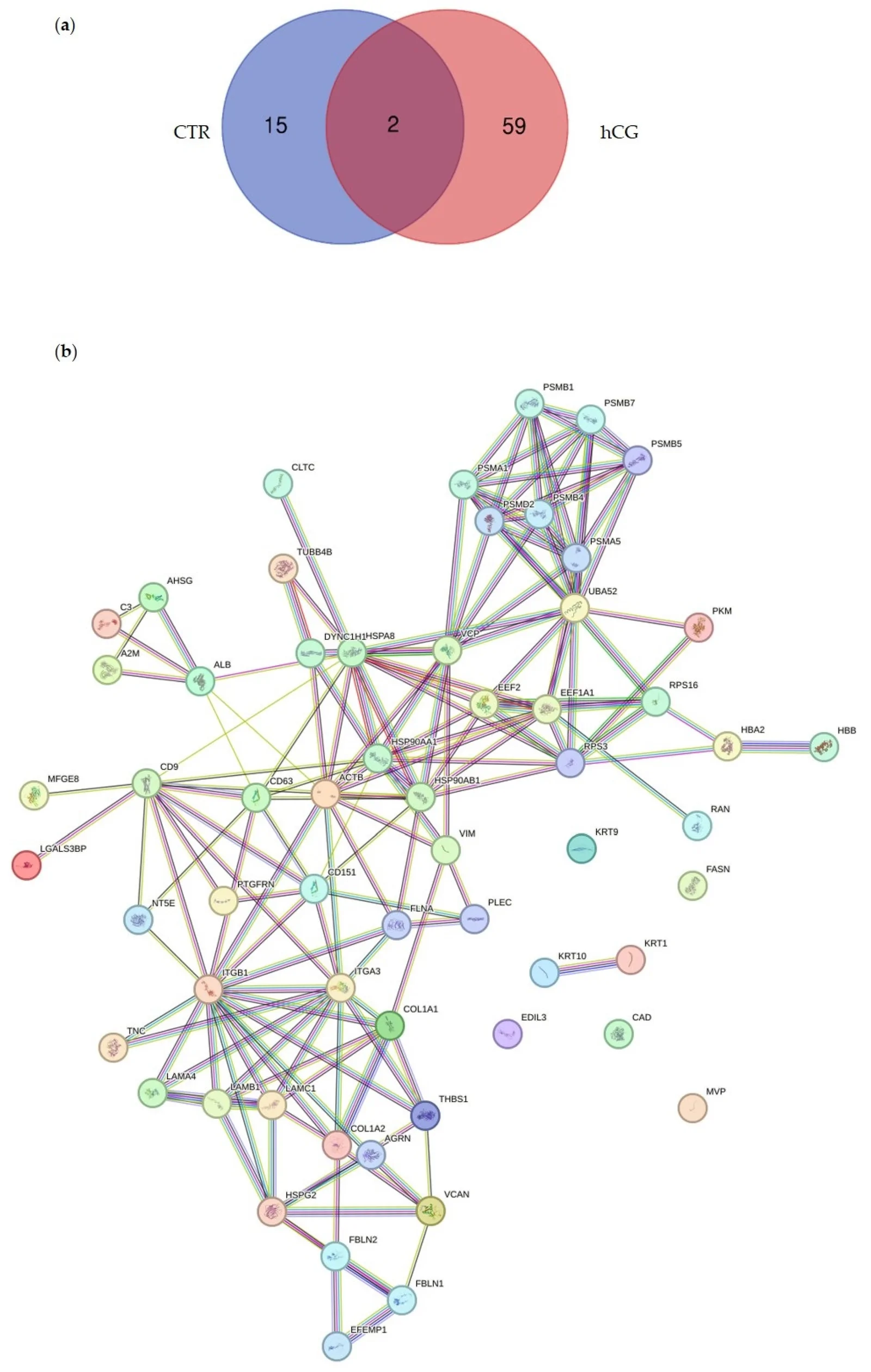
(**a**) Venn diagrams show the results of exosomal proteomic analysis comparing hCG-treated samples with the untreated control group (CTR). The diagrams illustrate the total number of proteins detected in each group, emphasizing both the shared proteins and those present uniquely in either the hCG-treated or control (CTR) group. (**b**) STRING network analysis shows the interaction map of exosome proteins exclusively found following hCG treatment. In the network, nodes represent individual proteins, and edges indicate predicted or known protein–protein interactions. Edge colors correspond to different sources of interaction evidence: green (neighborhood), red (fusion), blue (co-occurrence), yellow (text mining), purple (experimental), light blue (database), and black (co-expression).

Mass spectrometry-based qualitative proteomic analysis was conducted to identify the protein profiles of exosomes isolated from untreated and hCG-treated granulosa cells. As shown in Figure 2a, the Venn diagram illustrates the proteins found in exosomes derived from the hCG-treated samples compared to the untreated control (CTR). This analysis identified 15 proteins in the control group, 2 common proteins, and 59 proteins identified after hCG treatment. A comprehensive list of all identified proteins is provided in Supplementary Table S1.

Figure 2b shows the protein interaction network for proteins identified after gonadotropin treatment. This network was generated using STRING, and the thickness of the lines represents the strength of the interaction score.

Analysis of these proteins revealed several molecules involved in extracellular matrix signaling, steroidogenesis, and protein catabolism. Notably, integrins (-β1 and -α3) were identified, along with proteins that mediate intercellular communication and cell–matrix interactions, including Galectin-3-binding protein (LGALS3BP), Versican, and Thrombospondin-1.

Moreover, we found the tetraspanins CD63, CD9, and CD151, widely recognized as specific exosome markers, which are closely associated with integrins and other molecules such as LGALS3BP, forming complexes that facilitate intercellular communication, vesicle targeting, and the transfer of bioactive molecules to recipient cells. Furthermore, we identified fatty acid synthase (FASN), a multifunctional enzyme that catalyzes the de novo synthesis of long-chain saturated fatty acids and modulates steroidogenesis. Fatty acids produced by FASN, such as arachidonic acid, can activate key steroidogenic enzymes, including steroidogenic acute regulatory protein (StAR), which plays a crucial role in ovarian steroidogenesis during follicular development [28]. Finally, we identified several proteasome subunits, including α-1, α-5, and β-1, β-4, β-5, and β-7, which are components of proteasome complexes responsible for the proteolytic degradation of most intracellular proteins.

### 3.3. Gene Ontology (GO) and Reactome Pathway Enrichment Analysis

**Figure 3:**
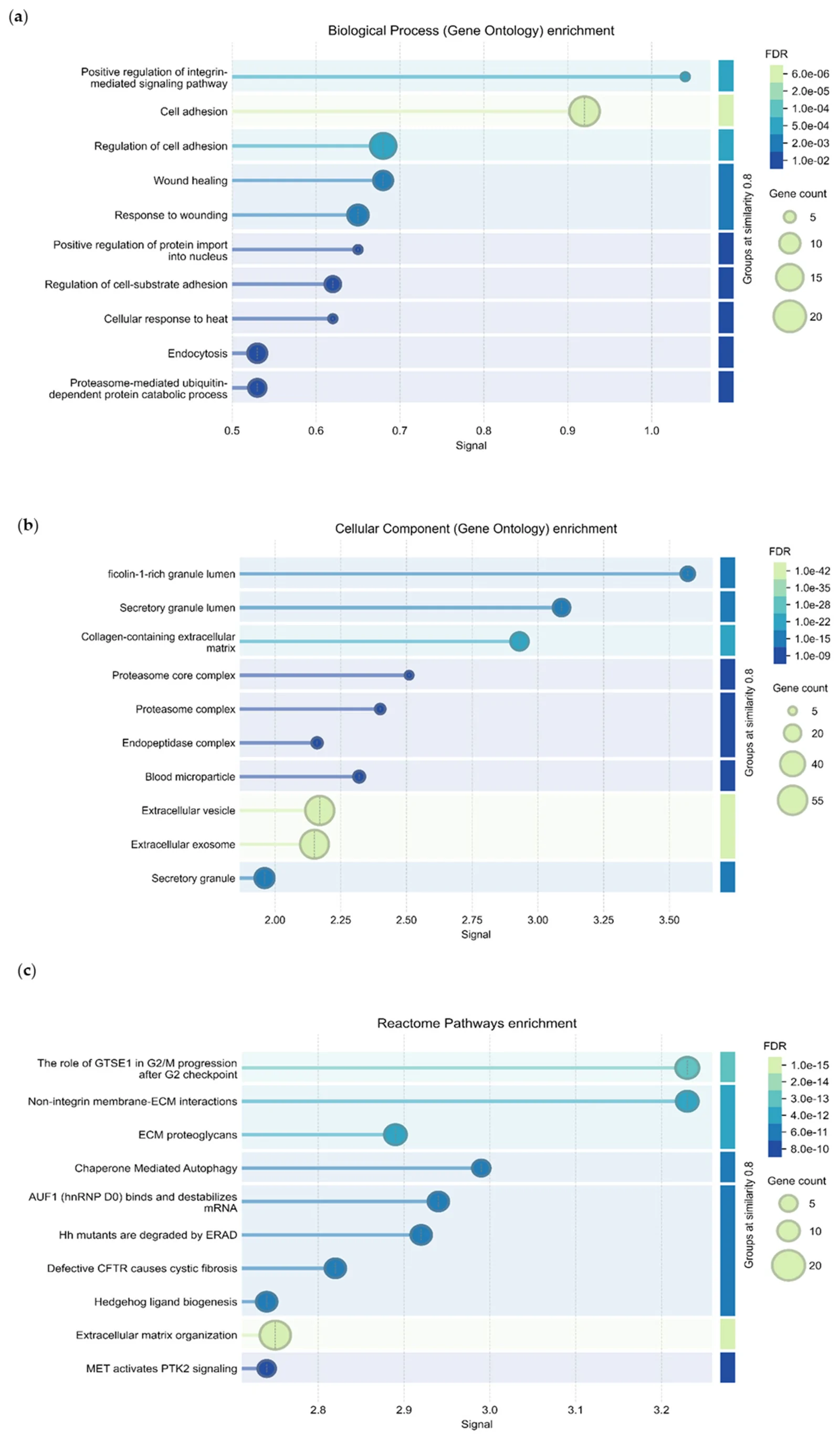
Gene Ontology (GO) enrichment analysis was conducted on exosomal proteins significantly modulated following hCG treatment, focusing on the Biological Process (**a**) and Cellular Component (**b**) categories. Bubble size represents the number of genes associated with each term, while color denotes the false discovery rate (FDR). (**c**) Pathway enrichment analysis of the 59 proteins uniquely identified in hCG-derived exosomes was performed using the Reactome database and ranked according to *p*-value.

Gene Ontology (GO) enrichment analysis of the differentially expressed genes revealed a significant overrepresentation of biological processes predominantly associated with cell adhesion and integrin-mediated signaling (Figure 3a). Among these, positive regulation of integrin-mediated signaling pathway emerged as the most enriched term, displaying the highest signal score and the lowest false discovery rate (FDR), and thereby highlighting an involvement of integrin-related signaling mechanisms. Processes associated with cell adhesion were also enriched, suggesting coordinated regulation of cell–cell and cell–matrix interactions. These pathways were supported by relatively high gene counts, highlighting their biological relevance in the analyzed gene set. In addition, enrichment of endocytosis and the proteasome-mediated ubiquitin-dependent protein catabolic process further suggests increased protein turnover and intracellular trafficking during these biological responses. Overall, the GO enrichment profile suggests a biological program characterized primarily by adhesion remodeling, integrin signaling, and repair-associated processes. Gene Ontology enrichment analysis for the Cellular Component category revealed that the identified genes were predominantly associated with extracellular, vesicular, and proteolytic compartments (Figure 3b). The most enriched term was ficolin-1-rich granule lumen, which showed the highest signal value and an extremely low FDR, indicating strong localization of the gene products to specialized secretory granule compartments.

Additional highly enriched terms included secretory granule lumen and secretory granule, further emphasizing the association of regulated secretory pathways. Enrichment of extracellular vesicle and extracellular exosome, supported by large gene counts, suggests a role for vesicle-mediated transport and intercellular communication. Genes were also associated with the collagen-containing extracellular matrix, indicating contributions to extracellular matrix organization or remodeling. In parallel, intracellular protein degradation machinery was represented by enrichment of the proteasome complex, proteasome core complex, and endopeptidase complex, highlighting active proteolytic processes. Overall, the Cellular Component enrichment profile suggests that the analyzed genes are mainly localized to secretory granules, extracellular vesicles, and proteasome-related complexes, consistent with coordinated roles in secretion, extracellular interactions, and protein turnover.

Reactome pathway enrichment analysis of exosome cargo following hCG treatment revealed modulation of pathways associated with cell cycle progression, extracellular matrix (ECM) remodeling, protein quality control, and signal transduction (Figure 3c). Enrichment was ranked by signal strength, with false discovery rates (FDRs) ranging from 1.0 × 10^−15^ to 8.0 × 10^−10^, indicating high statistical significance across all identified pathways.

The most strongly enriched pathway was “The role of GTSE1 in G2/M progression after G2 checkpoint”, suggesting enhanced representation of proteins involved in mitotic entry and checkpoint regulation within exosomal cargo. Similarly, pathways linked to non-integrin membrane–ECM interactions and ECM proteoglycans were significantly enriched, indicating that hCG treatment alters exosomal components related to extracellular matrix organization and cell–matrix communication.

Consistent with these findings, extracellular matrix organization displayed a high gene count and strong enrichment signal, highlighting substantial remodeling-associated cargo packaging. Additional enriched pathways included Chaperone-Mediated Autophagy, Hedgehog ligand biogenesis and MET activates PTK2, pointing to modulation of developmental and pro-migratory signaling pathways. Together, these findings were associated with an alteration, upon hCG treatment, of the composition of exosomal cargo, enriching pathways associated with cell proliferation, extracellular matrix remodeling, proteostasis, and growth factor signaling.

### 3.4. Comparative Analysis of Exosomal and Whole Secretome Proteomic Profiles

To determine whether hCG treatment differentially affects protein cargo in extracellular vesicles versus the total pool of secreted proteins, we compared the proteomic profile of KGN-derived exosomes with the global secretome previously characterized [12].

**Figure 4:**
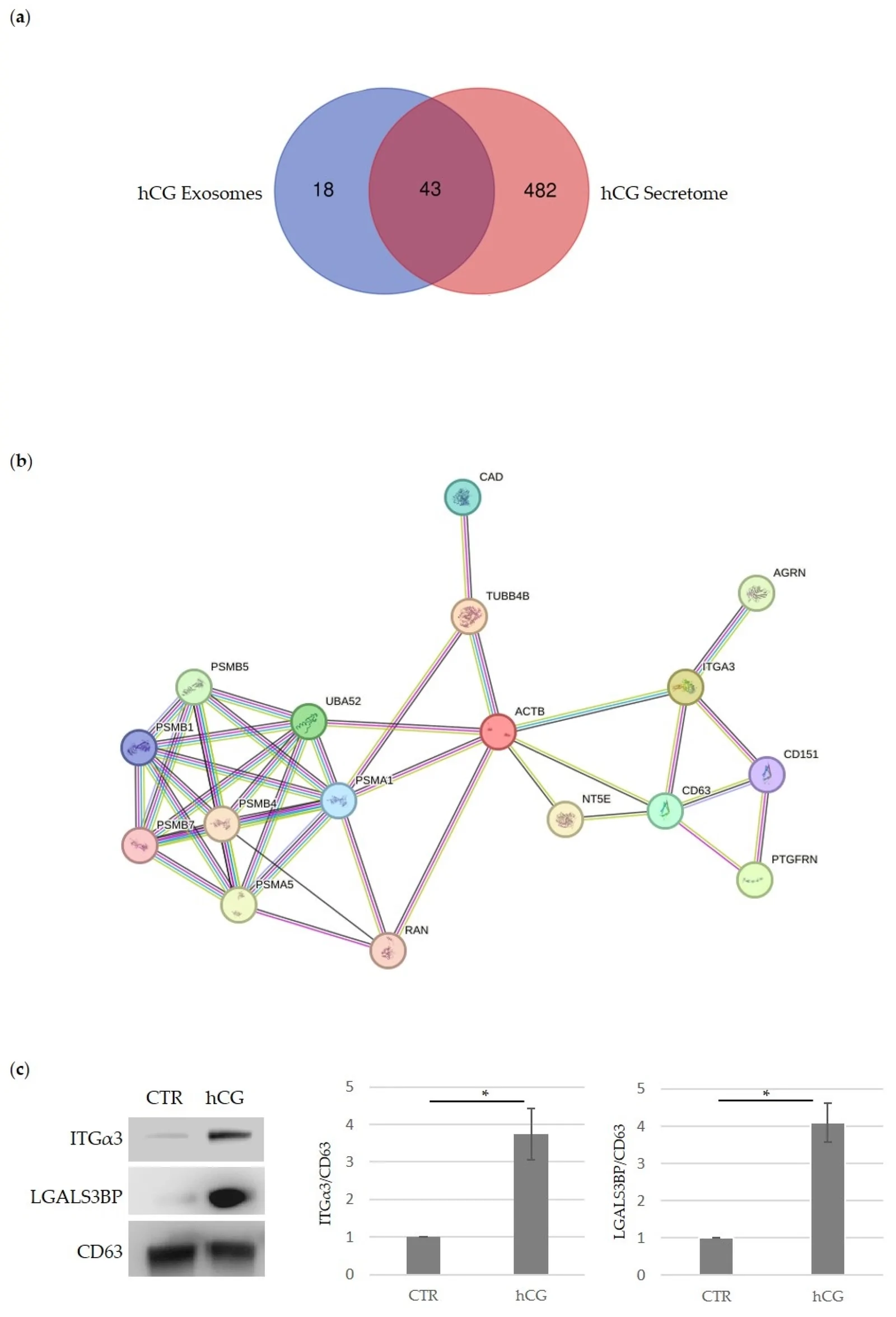
(**a**) Venn diagrams illustrate the results of the proteomic analysis comparing the KGN secretome and exosomal cargo following hCG treatment. The diagrams show the total number of proteins identified in each group, highlighting both the proteins shared between groups and those uniquely present in either the hCG-derived exosomes or the supernatant after hCG treatment. (**b**) STRING network analysis shows the interaction map of proteins exclusively found in exosomes following hCG treatment. In the network, nodes represent individual proteins, and edges indicate predicted or known protein–protein interactions. Edge colors correspond to different sources of interaction evidence: green (neighborhood), red (fusion), blue (co-occurrence), purple (experimental), yellow (text mining), light blue (database), and black (co-expression). (**c**) Representative Western blot analysis of ITGα3 and LGALS3BP expression in exosomes derived from KGN cells (untreated, CTR vs. hCG-treated). The histograms show the densitometric ratio of ITGα3 and LGALS3BP to CD63 (ITGα3/CD63 and LGALS3BP/CD63) (an exosomal marker used as loading control). The ITGα3/CD63 ratio in exosomes from untreated KGN cells (CTR) was set to 1. Data are presented as mean ± standard deviation (SD) from three independent biological replicates (* *p* < 0.05).

Figure 4 shows the qualitative proteomic analysis of proteins packaged into KGN-derived exosomes and those released into the culture medium following hCG stimulation. This analysis revealed that gonadotropin stimulation led to the secretion of 482 proteins into the conditioned medium, 18 proteins specifically identified in exosomes, and 43 proteins present in both fractions (Figure 4a). A comprehensive list of all identified proteins is provided in Supplementary Table S2.

A total of 482 proteins selectively secreted upon hCG treatment (Figure 4a) were mainly associated with cytoskeletal organization and extracellular matrix (ECM)-related pathways, as we previously reported [12]. Among these, semaphorin 3C, a modulator of cytoskeletal dynamics and integrin-mediated signaling [29], and Fibronectin-1 (FN1), a key ECM glycoprotein involved in cell proliferation, migration, and differentiation, were identified. Moreover, we found proteins, such as ezrin and moesin, that are also involved in cytoskeletal remodeling. This process is a key element in mediating the molecular trafficking of regulatory proteins, granulosa cell differentiation and the molecular signaling pathways involved in these events.

A total of 43 proteins were identified as common to both KGN-derived exosomes and the global secretome following hCG stimulation (Figure 4a). These shared proteins included several functional categories. A substantial proportion consisted of extracellular matrix and adhesion-related proteins, including collagens, laminin subunits, fibulins, Thrombospondin-1, tenascin, integrin β1, and heparan sulfate proteoglycan, indicating coordinated extracellular matrix remodeling through both soluble and vesicle-associated pathways. The common set also included cytoskeletal and structural proteins (keratins, vimentin, filamin-A, plectin, dynein), as well as proteins involved in vesicle trafficking and exosome biology, such as CD9 and clathrin heavy chain 1.

These data, revealing a partial overlap between exosomal cargo and the total secretome upon hCG treatment, suggest coordinated regulation of vesicular and non-vesicular protein secretion.

Figure 4b shows the protein interaction network (STRING) for the 18 proteins identified in exosomes following hCG treatment. Analysis of these proteins revealed several key molecules, including the tetraspanins CD63 and CD151, both of which are commonly enriched on the exosome surface. Furthermore, we identified Integrin α3 (ITGα3), which is a member of the integrin family of cell adhesion receptors, and is crucial for granulosa cell function and ovarian follicle development [30]. Moreover, as previously mentioned, we identified proteins with proteasomal activity, including the α- and β-subunits of the proteasome. Finally, we identified prostaglandin F2 receptor negative regulator (PTGFRN), a transmembrane protein whose principal binding partners are CD9 and CD81, members of the tetraspanin family that is implicated in diverse cellular processes, including adhesion, migration, cell differentiation, and protein trafficking [31].

These results, highlighting a selective distribution of proteins between exosomal and soluble fractions, suggest distinct mechanisms of protein release in response to gonadotropin stimulation.

To validate the proteomic data, the expression of Integrin α3 (ITGA3) and LGALS3BP in KGN-derived exosomes following hCG treatment was evaluated by Western blot. As illustrated in Figure 4c, Integrin α3 and LGALS3BP levels in exosomes were markedly elevated after 48 h of gonadotropin stimulation compared with exosomes from untreated control cells.

## 4. Discussion

In this study, we investigated whether hCG influences exosome production in KGN cells and performed a comprehensive qualitative proteomic analysis of their protein cargo. The exosomal profile was further compared with the total conditioned medium (secretome) following gonadotropin stimulation, aiming to identify proteins selectively enriched in exosomes that could reflect an effective granulosa cell response.

Although the KGN model does not fully recapitulate the complexity of the in vivo ovarian microenvironment, it represents a well-established and appropriate system for mechanistic studies, enabling controlled investigation of granulosa cell responses to gonadotropin stimulation. The KGN cell line retains key features of granulosa cells, including functional FSH receptor expression and steroidogenic activity [11]. More recently, it has also been applied to investigate extracellular vesicles in the ovarian context [15].

Our results, although qualitative, suggest that hCG may alter exosomal composition, leading to an enrichment of proteins involved in cellular differentiation, signal transduction, and intercellular communication. These exosomal factors may contribute to granulosa cell maturation and modulation of the ovarian microenvironment by facilitating paracrine signaling and intercellular cross-talk.

Among the proteins found in KGN-derived exosomes in response to hCG treatment we highlighted CD63 and CD151. These tetraspanins are highly enriched on exosomes and widely used as exosome markers, but they also actively regulate exosome biogenesis, cargo selection, and downstream biological effects [32]. It has been demonstrated that tetraspanins serve as pivotal organizers of membrane microdomains, shaping both the composition and functional dynamics of extracellular vesicles [33]. Their interactions with integrins and proteins like LGALS3BP promote the assembly of coordinated molecular complexes, which enhance vesicle stability and define their functional specificity [34]. These associations are crucial for the selective sorting of cargo and the precise delivery of extracellular vesicles to target cells. Consequently, tetraspanins act as essential regulators of exosome formation and activity, enabling efficient intercellular communication through the targeted transport of bioactive molecules.

Among these, CD63 plays a key role in directing cholesterol into intraluminal vesicles, thereby establishing a reservoir that is ultimately released through exosomes. When CD63 is absent, this trafficking pathway is disrupted, and cholesterol is instead rerouted away from endosomes, indicating that CD63 is essential for maintaining proper intracellular cholesterol distribution [35].

In the context of granulosa cell research, CD63 has been identified on extracellular vesicles (EVs) present in follicular fluid that are internalized by granulosa cells (GCs), where they regulate both steroidogenesis and gene expression. This highlights CD63^+^ EVs as important mediators of intrafollicular communication [15]. Varik and colleagues reported that large extracellular vesicles (LEVs) stimulate testosterone synthesis in GCs, whereas small extracellular vesicles (SEVs) do not affect steroid hormone secretion. However, SEVs induce widespread transcriptional changes in GCs, affecting pathways associated with transcriptional regulation, TGF-β signaling, extracellular matrix remodeling, and cell cycle progression [15].

Follicular fluid-derived extracellular vesicles (EVs) are central mediators of communication within the ovarian follicle [36]. By transferring bioactive molecules, they contribute to embryo development and support the expansion of the cumulus–oocyte complex through the regulation of key genes such as prostaglandin-endoperoxide synthase 2 (PTGS2), pentraxin-related protein 3 (PTX3), and tumor necrosis factor alpha-induced protein 6 (TNFα-IP6) [36]. These vesicles are also internalized by granulosa cells, highlighting a bidirectional signaling network within the follicular environment.

Recent research by Zhen Liu and colleagues compared the protein content of follicular fluid exosomes from younger and older women [37]. Although no differences were found in vesicle size or morphology, their proteomic profiles varied markedly. Exosomes from older women showed higher levels of proteins associated with immune activity, host–pathogen interactions, and metabolic imbalance, suggesting a potential link to age-related fertility decline. Functional analyses demonstrated that exosomes promote follicle maturation, particularly those derived from younger individuals, with proteins such as ENO1, HSP90B1, fetuin-B, C7, and APOC4 identified as key contributors [37]. These proteins were not detected in the exosomal cargo released by hCG-treated KGN cells in our study, likely reflecting the intrinsic limitations of the KGN in vitro model. As a granulosa-like tumor-derived cell line, KGN cells cannot fully reproduce the complexity and cellular heterogeneity of the in vivo follicular microenvironment, where follicular fluid exosomes originate from multiple ovarian cell types, including mural and cumulus granulosa cells, theca cells, immune cells, and possibly oocyte-derived vesicles. By contrast, our study specifically analyzed exosomes secreted by a single granulosa-like cell population under controlled hCG stimulation conditions. While EVs present in follicular fluid have been widely recognized as key mediators of communication between the oocyte and surrounding somatic cells, our findings demonstrate that granulosa cells themselves actively release exosomes following gonadotropin stimulation. These results suggest that granulosa cell-derived exosomes likely represent an additional and significant signaling component within the follicular microenvironment and may contribute to the molecular cross-talk required to support proper follicular growth and development.

Protein enrichment analyses of KGN-derived exosomes suggest that hCG stimulation could be associated with coordinated cellular response characterized by remodeling, intercellular communication, and adaptation. Prominent processes include integrin-mediated signaling and cell adhesion, pointing to dynamic regulation of cell–extracellular matrix interactions, a hallmark of tissue remodeling and migration [38]. Moreover, the identified proteins are predominantly associated with secretory compartments, particularly extracellular vesicles and exosomes, underscoring their central role in mediating cell-to-cell communication. Pathway analysis of the exosomal cargo further reveals enrichment in proteins involved in cell cycle progression, extracellular matrix organization, and key signaling pathways linked to proliferation and migration. Together, these findings lead to the hypothesis that hCG drives a proliferative, pro-remodeling phenotype, with exosomes acting as active modulators of recipient cells and contributing to complex adaptive responses.

The comparative analysis between the exosomal proteome and the secretome provides insight into how hCG stimulation differentially regulates protein release through vesicular and non-vesicular pathways. The identification of a large number of proteins secreted into the conditioned medium, alongside a much smaller subset specifically enriched in exosomes, suggests that hCG does not simply increase global secretion but instead promotes selective sorting mechanisms that determine the protein destination.

Consistent with previous observations [12], the enrichment of cytoskeletal and ECM-related proteins within the soluble secretome supports the idea that hCG drives structural and functional remodeling of granulosa cells. Proteins such as semaphorin 3C and fibronectin-1 point toward active reorganization of the cytoskeleton and enhanced cell–matrix interactions. These processes are essential for granulosa cell differentiation and may facilitate changes in cell shape, motility, and signaling capacity in response to gonadotropin stimulation [13].

The subset of proteins shared between exosomes and the total secretome further supports the idea of coordinated extracellular remodeling. The presence of ECM components (e.g., collagens, laminins, and proteoglycans) and adhesion molecules in both fractions suggests that cells employ complementary mechanisms—soluble secretion and vesicle-mediated delivery—to modulate the extracellular environment. At the same time, the inclusion of cytoskeletal and vesicle trafficking proteins in this shared pool highlights the tight coupling between intracellular structural dynamics and extracellular communication.

Notably, the proteins enriched in exosomes define a more specialized and functionally targeted cargo. The presence of canonical exosomal markers such as CD63 and CD151, together with proteins such as integrins, agrin, and prostaglandin F2 receptor negative regulator (PTGFRN), indicates that these vesicles are specifically equipped to mediate cell adhesion, migration, and signal transduction in recipient cells. Additionally, the presence of enriched proteasome subunits within exosomes is especially noteworthy, as it suggests a potential role for extracellular vesicles in modulating proteostasis beyond the cell of origin, possibly influencing protein turnover in neighboring cells.

Among these proteins uniquely identified in granulosa cell-derived exosomes upon hCG treatment, we highlighted Integrin-alpha3 (ITGα3) and LGALS3BP, whose presence was further confirmed by Western blot analysis.

Integrins, including alpha subunits, are cell adhesion receptors that mediate interactions between cells and the extracellular matrix, playing crucial roles in reproductive processes [39]. Furthermore, it has been shown that integrins are essential for the communication between germ cells and surrounding tissues during ovulation [30,40]. In animal models, disruption of β -integrin or its associated proteins impairs ovulation, highlighting the importance of integrin-mediated cell–extracellular matrix (ECM) interactions [41]. These interactions regulate ovulation through phosphatidylinositol-3 (IP3) signaling pathways, and defects can be partially rescued by enhancing IP3 signaling, underscoring integrins’ regulatory role in fertility [38]. Recent studies have demonstrated that integrins are not only present on the cell surface but are also incorporated into exosomes, where they have emerged as key regulators of multiple biological processes [42,43]. In particular, they are recognized for their ability to direct the tissue-specific distribution of exosomes, enhance their recognition and uptake by recipient cells, and promote the transfer of membrane-associated proteins and kinases to target cells, thereby amplifying downstream signaling events [44,45]. Among them, integrins, such as ITGα3, contribute to intercellular communication by transferring bioactive molecules that modulate signaling pathways in recipient cells, thereby influencing key cellular behaviors including migration, invasion, and resistance to apoptosis [46].

Furthermore, we highlighted the prostaglandin F2 receptor negative regulator (PTGFRN). Several studies show that granulosa cells depend strongly on prostaglandin signaling pathways, particularly involving the prostaglandin F2α receptor (PTGFR), the cyclooxygenase enzyme PTGS2, and various non-coding RNAs that modulate these regulatory elements [45,46,47,48,49]. Although PTGFRN has been shown to influence PGF2α signaling in other tissues, its specific function in granulosa cells remains unclear. Intriguingly, Marquez and colleagues showed that PTGFRN is a transmembrane protein that physically associates with the tetraspanins CD9, CD81, and CD151, as well as integrin, forming part of the “tetraspanin web” that coordinates a multitude of cellular processes such as adhesion, cell differentiation, signaling, and trafficking [31,50].

Overall, these findings lead to the hypothesis of a model in which hCG stimulation induces a dual secretory response: a broad release of proteins involved in ECM remodeling and cytoskeletal reorganization into the extracellular space, and a more selective packaging of signaling-competent proteins into exosomes. This selective distribution underscores the existence of distinct but coordinated secretion pathways and highlights the role of exosomes as active conveyors of specific biological signals, rather than passive byproducts of secretion.

Most investigations into hCG’s action in granulosa cells have primarily centered on gene expression, chromatin remodeling, and the soluble secretome, while comparatively little attention has been directed toward granulosa cell-derived exosomes [12,51,52]. Nonetheless, placing these findings within the broader context of exosome biology offers a useful conceptual framework for interpreting how hCG-induced cellular reprogramming may be harnessed in clinical practice.

Various strategies for inducing final follicular maturation have been described, differing in both timing and hormonal composition. Although the physiological trigger involves endogenous LH and FSH, hCG is widely used in controlled ovarian stimulation cycles. More recently, the “dual” or “double” trigger—combining GnRH agonist-induced LH and FSH surges with hCG administration—has gained attention. This approach has been associated with increased expression of genes involved in steroidogenesis (e.g., StAR, CYP19) and oocyte maturation (e.g., COX2, Amphiregulin) compared with both the natural LH+FSH surge and hCG alone [53], supporting its clinical advantage.

Despite these advances, direct evidence linking hCG-specific cellular reprogramming to changes in the content of granulosa cell-derived exosomes remains limited. The present report therefore provides an exploratory, hypothesis-generating perspective on how hCG stimulation may influence exosomal cargo. Future studies should aim to systematically compare exosomal profiles across hCG-only, dual/double trigger, and natural LH+FSH conditions. Elucidating these relationships represents a promising yet underexplored opportunity to refine and personalize assisted reproduction protocols.

## 5. Conclusions

Our data provide an exploratory and hypothesis-generating overview of potential changes in exosomal cargo following hCG stimulation, revealing selective enrichment of proteins involved in ECM remodeling, integrin signaling, steroidogenesis, and proteostasis.

Limitations of our study include that it relies on in vitro granulosa cell cultures, which may not fully replicate the in vivo ovarian microenvironment. Future studies using quantitative proteomic approaches will be necessary to validate our findings. Moreover, it would be valuable for future investigations to integrate functional validation and analyses across different follicular stages to establish the physiological relevance of these exosome-mediated pathways. Collectively, these data reveal the critical role of exosome-mediated communication in ovarian function and provide a basis for future mechanistic research and potential clinical interventions.

## Data Availability

The original contributions presented in this study are included in the article/Supplementary Materials. Further inquiries can be directed to the corresponding author.

## Supplementary Materials

The following supporting information can be downloaded at: https://www.mdpi.com/article/10.3390/cells15110956/s1, Figure S1: Calnexin expression in exosomes and total lysates from KGN cells. Table S1. Proteomic analysis of differentially expressed exosomal proteins. Table S2. Comparative Analysis of Exosomal and Whole Secretome Proteomic Profiles.
