# Supplementary material for: Human Chorionic Gonadotropin (hCG)-Induced Remodeling of the Granulosa Cell Exosomal Proteome: Implications for Follicular Communication
Source: Cells. 2026 May 22;15(11):956. doi: 10.3390/cells15110956 (PMC13256384; doi:10.3390/cells15110956)
Supplement: Supplementary file 1 [file cells-15-00956-s001.zip › cells-4317266-Suppl fig S1-revised.pdf]

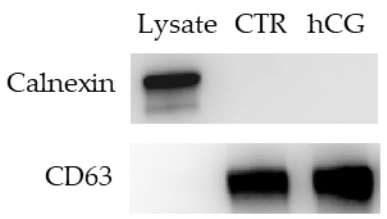

Supplementary Figure S1 : Rapresentative western blot of calnexin and CD63 in KGN cell lysates and exosomes from KGN untreated (CTR) or hCG treated.
